# UVA Irradiation of Dysplastic Keratinocytes: Oxidative Damage *versus* Antioxidant Defense

**DOI:** 10.3390/ijms131216718

**Published:** 2012-12-06

**Authors:** Marina T. Nechifor, Cristina M. Niculiţe, Andreea O. Urs, Teodor Regalia, Mihaela Mocanu, Alexandra Popescu, Gina Manda, Diana Dinu, Mircea Leabu

**Affiliations:** 1 Faculty of Biology, University of Bucharest, Bucharest 050095, Romania; E-Mails: nemar_59@yahoo.com (M.T.N.); diana_dinu2006@yahoo.com (D.D.); 2 “Victor Babes” National Institute of Pathology, Bucharest 050096, Romania; E-Mails: cristina_niculite@yahoo.com (C.M.N.); aurs.bv@gmail.com (A.O.U.); tedyregalia@yahoo.com (T.R.); miha_moc@yahoo.com (M.M.); alessia.popescu@yahoo.com (A.P.); gina.manda@gmail.com (G.M.); 3 “Carol Davila” University of Medicine and Pharmacy, Bucharest 050096, Romania

**Keywords:** dysplastic keratinocyte, UVA, oxidative stress, membrane integrity, lipid peroxidation, LDH release, catalase

## Abstract

UVA affects epidermal cell physiology in a complex manner, but the harmful effects have been studied mainly in terms of DNA damage, mutagenesis and carcinogenesis. We investigated UVA effects on membrane integrity and antioxidant defense of dysplastic keratinocytes after one and two hours of irradiation, both immediately after exposure, and 24 h post-irradiation. To determine the UVA oxidative stress on cell membrane, lipid peroxidation was correlated with changes in fatty acid levels. Membrane permeability and integrity were assessed by propidium iodide staining and lactate dehydrogenase release. The effects on keratinocyte antioxidant protection were investigated in terms of catalase activity and expression. Lipid peroxidation increased in an exposure time-dependent manner. UVA exposure decreased the level of polyunsaturated fatty acids, which gradually returned to its initial value. Lactate dehydrogenase release showed a dramatic loss in membrane integrity after 2 h minimum of exposure. The cell ability to restore membrane permeability was noted at 24 h post-irradiation (for one hour exposure). Catalase activity decreased in an exposure time-dependent manner. UVA-irradiated dysplastic keratinocytes developed mechanisms leading to cell protection and survival, following a non-lethal exposure. The surviving cells gained an increased resistance to apoptosis, suggesting that their pre-malignant status harbors an abnormal ability to control their fate.

## 1. Introduction

Solar ultraviolet radiations damage human skin by targeting cellular and molecular events because of a deep penetration of the epidermis and dermis. The harmful effects of UVA (a low UV energy domain, 315–400 nm) include photoaging [1–3], carcinogenesis [4–7] or sunburns [8]. The deleterious effects of UVA radiation have been related to the oxidative stress generated by reactive oxygen species (ROS), as a consequence of photon absorption by endogenous target molecules [9,10]. There are many molecules in cells acting as potential chromophores for the absorption of UVA radiation. Some of them are compounds carrying unsaturated bonds, such as quinones, steroids, flavins, free porphyrins and heme-containing proteins [11,12]. The skin is extensively exposed to the ravage of ROS, due to its direct interaction with oxygen and sunlight on one hand, and, on the other hand, to its compounds, which absorb light and act as photosensitizers. The main cellular targets of ROS are DNA, proteins and membrane lipids [13]. The ROS generated by UVA radiation include singlet oxygen, superoxide, hydrogen peroxide and hydroxyl radical [14–16]. It is well established that these ROS not only exert oxidative damage, but may also play a central role in oxidative stress signaling pathways leading to cellular damage/apoptosis or adaptive responses [17–19]. Therefore, it may be thought that the antioxidant cell status could be critical in regulating cell behavior [20]. The cellular antioxidant defense mechanisms in the skin are both enzymatic and non-enzymatic [21–23]. Several enzymatic systems detoxifying ROS in the skin include catalase, glutathione peroxidase/reductase and superoxide dismutase. Deficiencies in these antioxidant systems cause ROS accumulation within the cell, inducing a sustained oxidative stress [24,25]. In addition to the constitutive antioxidants, the oxidative stress results in the upregulation of the enzymatic inducible antioxidants. One of the main inducible responses to UVA radiation is the oxidative induction of heme oxygenase-1 [26,27], and the enzymatic product of heme oxidative degradation, biliverdin/bilirubin, acts as a physiological antioxidant protecting the cells against lipid peroxidation [28]. All the above effects were observed in both normal and pathological cells, including keratinocytes, while less information exists regarding cells in a precancerous state and their ability to adapt or not to environmental stress.

Our study is focused on changes induced by the UVA radiation at the cell membrane level in cultured dysplastic keratinocytes, which represents the first target of UVA photons. Effects on both cell lipid biochemistry (in terms of lipid peroxidation and fatty acid composition changes) and cell membrane integrity and permeability were assessed. We have also been interested in understanding how the plasma membrane changes affect the cell’s decision to demise or to mount an adaptive response. In order to understand the role of antioxidant protection in the survival of UVA-exposed keratinocytes, we have investigated the activity and expression level of catalase and the consequences of the targeted inhibition of catalase. Our results support the idea that the dysplastic keratinocytes prove the ability to survive under oxidative stress generated by UVA exposure by eliciting adaptive/reparatory mechanisms related to the protection of membrane composition, permeability and integrity.

## 2. Results and Discussion

Most of the studies regarding the effects of UVA radiation on mammalian cells have focused on DNA damage and repair [29–32], mutagenesis [33,34] and carcinogenesis [4,35,36]. However, there is accumulating evidence that UVA can also affect the plasma membrane structure and function [37–40]. Our study aimed to assess the effects of UVA on cell membrane and antioxidant protection in dysplastic keratinocytes.

### 2.1. Effects of UVA on Keratinocyte Cell Membrane

#### 2.1.1. Cell Membrane Biochemistry

**Figure 1:**
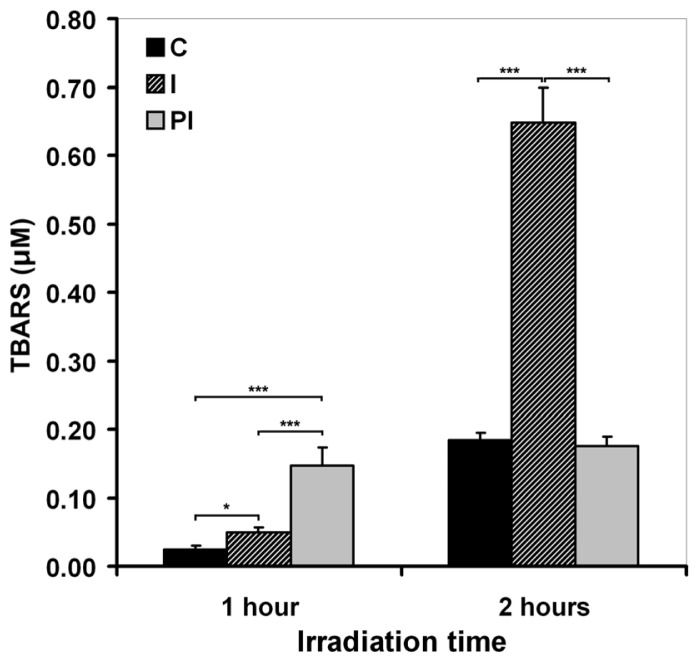
The level of TBARS released in the medium by keratinocytes exposed to UVA depends on irradiation time. C—mock-irradiated, I—just after irradiation, PI—24 h post-irradiation culture. Bars represent average values ± SD, for five experiments; ^*^p < 0.05; ^***^p < 0.001.

It has been suggested that ROS generated as a consequence of UVA exposure of cells affect the physicochemical properties of the membrane [41]. Our results proved that irradiation of DOK cells leads to lipid peroxidation resulting in TBARS release in the medium (Figure 1). No significant effect was noted after irradiation times shorter than 1 h (not shown). The level of the released TBARS in the medium was assessed both immediately after 1 h or 2 h sessions of UVA irradiation, and at 24 h post-irradiation. We noted an increase of TBARS level immediately, after both sessions of irradiation: a 1.5-fold increase after 1 h exposure, and a four-fold increase after 2 h exposure (Figure 1). On the other hand, the TBARS level registered in the culture medium at 24 h post-irradiation was about 5 times higher after 1 h irradiation, compared to a 2 h exposure session. It is noteworthy that in the case of 2 h irradiation, after a strong increase shown right after UVA exposure, the level of TBARS dramatically diminished in the medium of cells cultured for 24 h post-irradiation (Figure 1). The results suggest a different kinetics in lipid peroxidation induced by UVA as a function of irradiation time and, therefore, as a function of the amount of radical species initiating the process.

**Figure 2:**
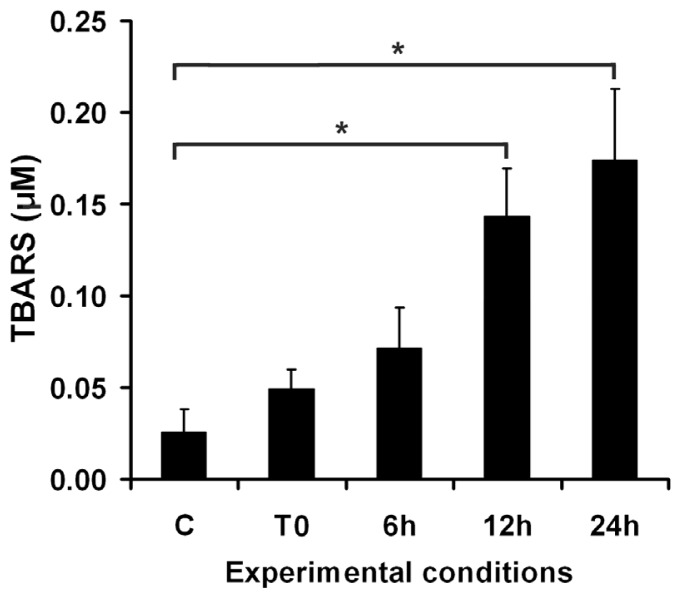
Dynamics of TBARS detected in the culture medium of DOK cells at different time intervals post-irradiation. C—mock-irradiated, T0—just after irradiation, 6 h—TBARS level in a 6 h post-irradiation DOK cell culture, 12 h—TBARS level in a 12 h post-irradiation cell culture, 24 h—TBARS in culture medium in a 24 h post-irradiation cell culture. Bars represent average values ± SD, for three experiments; ^*^p < 0.05.

In order to understand the dynamics of the lipid peroxidation process in DOK cell culture, the release of TBARS was monitored immediately after a 1 h irradiation session, and at different time intervals post-irradiation (Figure 2). The results show a progressive increase in the TBARS release during the 24 h post-UVA exposure, thereby suggesting the propagation of the chain peroxidation process.

It is noteworthy that the persistence of lipid peroxidation products in culture medium depends on the UVA exposure time, as evidenced at 24 h post-irradiation. To explain the pattern suggesting a different kinetics in TBARS level changes, two important issues have to be pointed out: (i) lipid peroxidation is a chain process depending on the level of initiating free radicals, and (ii) lipid peroxidation end-products do not accumulate, undergoing complex transformations. Therefore, after a 1 h session of UVA irradiation, the chain reaction is started, and the subsequent and final stages occur even at 24 h post-irradiation. The higher number of free radicals generated into the system, as a consequence of a longer UVA exposure, is translated into an accelerated kinetics of the process. Therefore, even the late phase of decomposition of the lipid hydroperoxides ends faster, *i.e.*, sooner than 24 h post-irradiation. Moreover, the end-products generated by the breakdown of lipid hydroperoxides, such as TBARS, may undergo further transformation. Similar results were previously reported for other types of human skin cell cultures, such as normal keratinocytes and fibroblasts [20,42–44]. Therefore, it seems that there is no difference in the UVA effects on normal cells (keratinocytes or fibroblasts) and dysplastic keratinocytes, in terms of lipid peroxidation.

**Figure 3:**
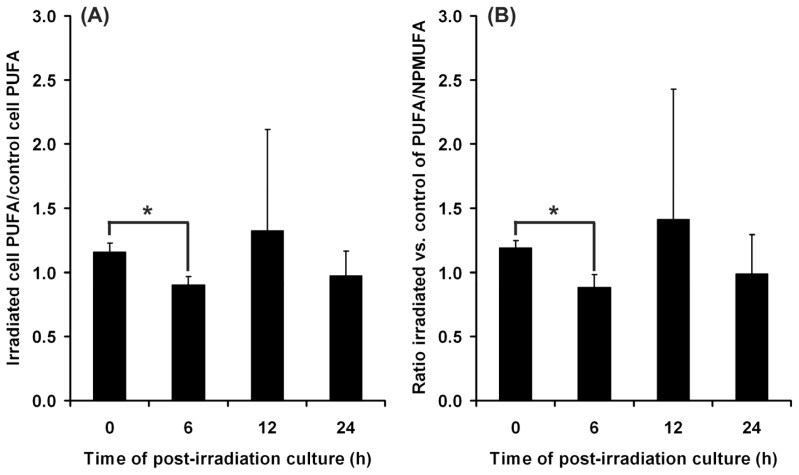
Changes induced by UVA irradiation on the fatty acid content of cellular phospholipids seem to be reversible. (**A**) the ratio sample/control of polyunsaturated fatty acid level at various time intervals post-irradiation, after UVA exposure; (**B**) the ratio peroxidizable/non-peroxidizable fatty acids in UVA irradiated versus mock-irradiated DOK cells. Bars represent average values ± SD, for five experiments; PUFA—polyunsaturated fatty acids; NPMUFA—non-peroxidizable saturated and mono-unsaturated fatty acids; ^*^p < 0.05.

Polyunsaturated fatty acids represent a target of the lipid peroxidation process. Therefore, we investigated the changes of their content in cell lipids immediately after irradiation and post-irradiation at various time intervals (Figure 3). The level of polyunsaturated fatty acids was found to decrease significantly at 6 h post-irradiation, and then, it gradually returned toward the initial level, as evidenced by the values recorded at 12 h and 24 h, respectively (Figure 3A). A similar dynamics was obtained by examining the ratio polyunsaturated *vs*. non-peroxidizable fatty acids (mono-unsaturated + saturated). This ratio showed a significantly decreased value at 6 h post-irradiation, and a return to the initial level at longer post-irradiation times (Figure 3B). These results suggest the ability of the cells to trigger a protective response against UVA effects. Therefore, even though polyunsaturated fatty acids are affected by peroxidation after UVA exposure, and TBARS remains detectable 24 h post-irradiation, the level of peroxidizable fatty acids starts to be restored earlier.

#### 2.1.2. Cell Membrane Integrity and Permeability

**Figure 4:**
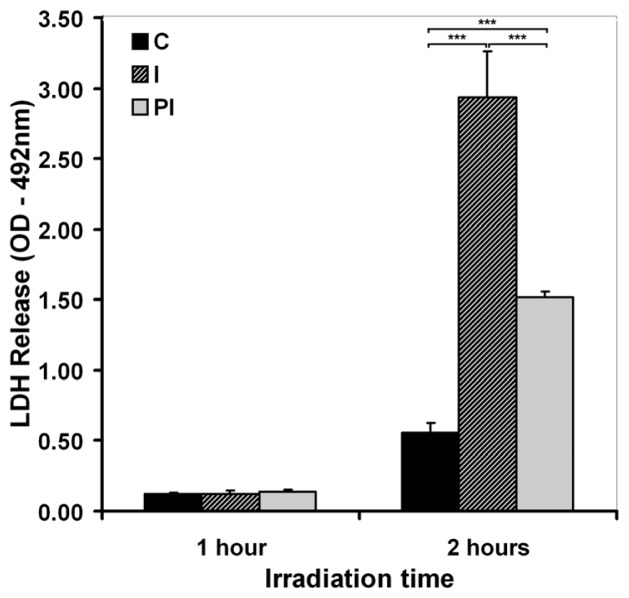
The effect of irradiation time on cellular membrane integrity, assessed by LDH release in cell culture medium. C—mock-irradiated, I—just after irradiation, PI—24 h post-irradiation culture. Bars represent average values ± SD, for five experiments; ^***^p < 0.001.

The peroxidation of membrane lipids obviously affects membrane integrity. For that reason, the increase of TBARS level in the culture medium of irradiated DOK led us to analyze the extent of cell lysis in response to the UVA exposure. A session of 1 h UVA exposure that induced a slight occurrence of lipid peroxidation did not affect membrane integrity in terms of LDH release following cell lysis (Figure 4). Conversely, after 2 h irradiation, a substantial release of LDH was registered, indicating a large extent of lysis (about 5-fold increase just after irradiation, and about 2.5-fold increase at 24 h post-irradiation). Our assumption that lipid peroxidation affects the membrane integrity was confirmed by the significant value of Pearson’s correlation coefficient (*r* = 0.995) between TBARS levels and the extent of cell lysis. This means that the oxidative stress mechanisms elicited by UVA exposure affect the membrane integrity, resulting in cell lysis.

**Figure 5:**
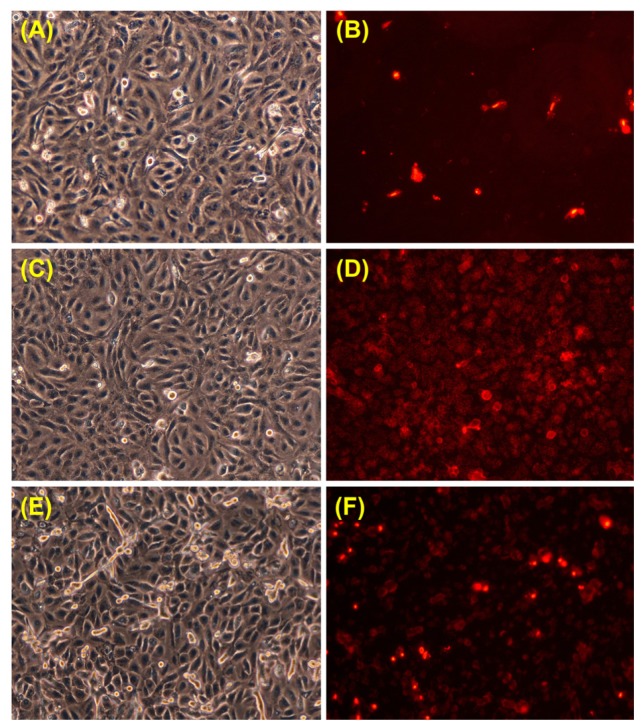
Phase-contrast (left) and propidium iodide fluorescence (right) images for DOK cells in culture before irradiation (**A**, **B**), immediately after a 1 h session of UVA irradiation (**C**, **D**) and in a 24 h post-irradiation culture (**E**, **F**).

It is known that UVA radiation affects the membrane at the molecular level by oxidative changes in lipids and proteins [42,45,46]. Therefore, membrane permeability is expected to undergo changes as a result of UVA exposure of keratinocytes. Propidium iodide is one of the markers extensively used in membrane permeability evaluation. We examined the propidium iodide staining of keratinocytes in the experimental conditions where the level of membrane lysis was negligible (*i.e.*, 1 h UVA exposure), both immediately after irradiation, and 24 h post-irradiation (Figure 5). The number of propidium iodide-stained cells was significantly higher immediately after UVA exposure, suggesting an increase in plasma membrane permeability (Figure 5D). The number of propidium iodide-stained cells decreased 24 h post-irradiation (Figure 5F), indicating the ability of the cells to restore the barrier function of the membrane, but it still remained greater than the one registered for control cells. The results suggest that 1 h exposure of keratinocytes to UVA modifies the membrane permeability, at least for small molecules, with no effects on membrane integrity in terms of cell lysis, as shown by the LDH release dynamics (Figure 4).

Our data indicate that UVA-induced peroxidation in DOK cells affects plasma membrane integrity and/or permeability. One may presume that the consequences of lipid peroxidation on cell membrane are more complex, affecting membrane functionality and membrane-cytoskeleton interactions, since a number of proteins that stimulates the nucleation and regulation of actin filaments are activated/inactivated by certain plasma membrane phospholipids [47].

### 2.2. Effects of UVA on Catalase Activity and Expression

The reaction of mammalian cells to several noxious agents was studied extensively, two different responses being reported: cell death and active mounting of a protective response. Therefore, we were interested in investigating a putative adaptive response, in terms of enzymatic defense activity, triggered by exposure of DOK cells to UVA, and the induced oxidative stress. Such a defense mechanism includes the catalase, an antioxidant enzyme involved in the degradation of hydrogen peroxide into water and oxygen. Thus, we investigated how UVA exposure modulated the activity and the expression of this enzyme.

#### 2.2.1. UVA Effect on Catalase Activity

**Figure 6:**
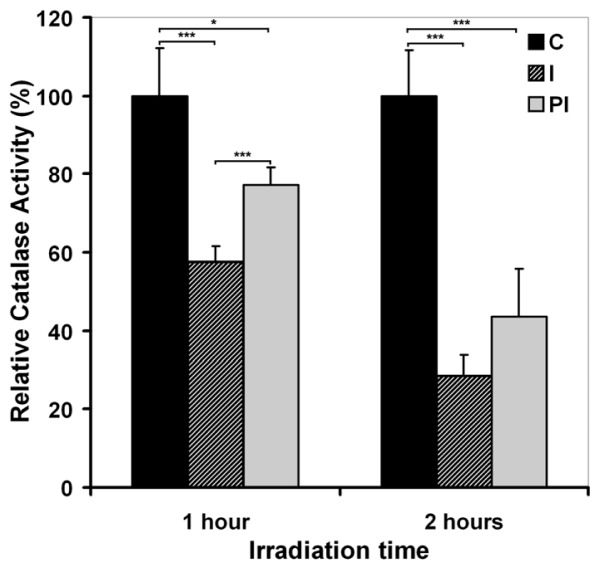
The effect of UVA irradiation on catalase activity immediately after 1 or 2 h sessions of irradiation (I) and 24 h post-irradiation (PI). Bars represent the percent of catalase activity relative to control (average ± SD, for 3 experiments); ^*^p < 0.05; ^***^p < 0.001.

Catalase activity was measured immediately after 1 and 2 h sessions of irradiation, and at 24 h post-irradiation. A significant difference in the level of enzyme activity was observed for irradiated cells compared to the control cells (Figure 6). A significant decrease in catalase activity was registered in the irradiated cells immediately after both 1 and 2 h of UVA exposure (by about 42% and about 71%, respectively). For the irradiated cells maintained in culture for 24 h post-irradiation, a restoration of the catalase activity was noted (up to about 79% for 1 h irradiated cells and about 44% for the 2 h irradiated cells).

The results demonstrate a decrease in catalase activity as an effect of UVA exposure and the tendency of the irradiated cells to restore the antioxidant activity of catalase in an exposure time-dependent manner.

It has previously been shown that hydrogen peroxide is an important redox active species involved in the deleterious effects of UVA radiation on the lipids and proteins of human skin cells [42]. Catalase represents the first line of defense against hydrogen peroxide mediated damage in all organs and tissues [48]. Catalase activity level in DOK cells exposed to UVA has diminished in our experimental conditions, depending on the exposure time. It is commonly accepted that catalase photoinactivation occurs through an oxygen-dependent photooxidation of the heme groups that involve ROS [49–51]. In addition, UVA-mediated oxidative modification of the catalase apoprotein, leading to structural alterations and catalytic deactivation, were previously shown [52,53]. The initial decrease of the activity noted by us may be considered as a direct effect of UVA causing catalase photoinactivation, while the restoring increase registered after a 24 h post-irradiation culture of the UVA-exposed cells may be considered a protective response in the attempt to re-establish a basal level of catalase activity (Figure 6). Similar data regarding the UVA-induced inactivation of catalase have previously been reported in cultured human skin fibroblasts [21] and in epidermal reconstructs made with keratinocytes or with keratinocytes and melanocytes derived from epidermal biopsies [53].

#### 2.2.2. UVA Effect on Catalase Expression

**Figure 7:**
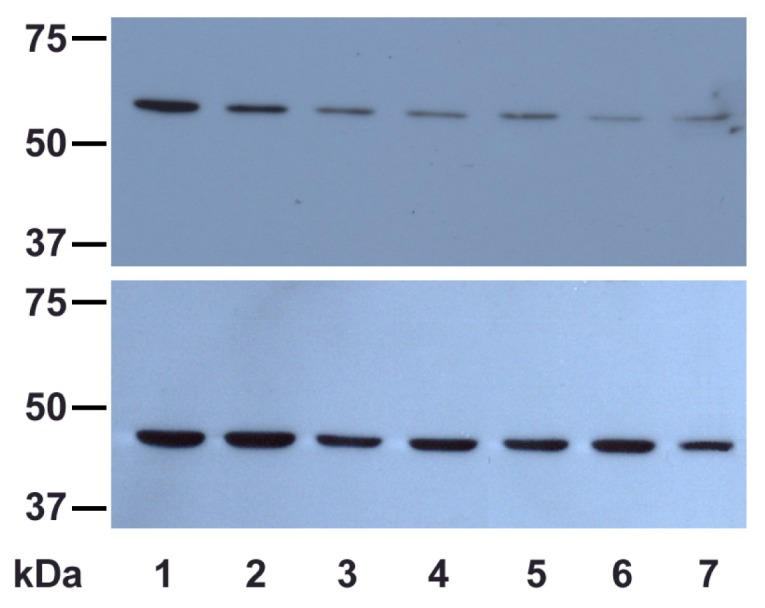
The effects of different UVA exposure times on the expression level of catalase in DOK cells (top) at various post-irradiation time intervals; β-actin loading control (bottom). 1—mock-irradiated cells; 2—irradiated cells (30 min exposure) just after irradiation; 3—irradiated cells (1 h exposure) just after irradiation; 4—irradiated cells (2 h exposure) just after irradiation; 5—irradiated cells (30 min exposure) at 24 h post-irradiation; 6—irradiated cells (1 h exposure) at 24 h post-irradiation; 7—irradiated cells (2 h exposure) at 24 h post-irradiation.

Despite the fact that catalase activity increased at 24 h post-irradiation, the expression level is significantly decreased compared to the control cells and even to the irradiated cells immediately after exposure, as evidenced by western blot analysis (Figure 7). This means that the inactivated catalase was continuously degraded (perhaps by using the heme metabolites as antioxidant defenders), while the *de novo*-produced catalase is more effective. Our results suggest a dual role for catalase in cellular antioxidant mechanisms as both an enzyme metabolizing the hydrogen peroxide and possibly as a source of antioxidant compounds provided by its heme prosthetic group released and modified after catalytic photo-inactivation induced by UVA.

### 2.3. Effects of UVA Exposure and Catalase Inhibition on Keratinocyte Fate

To test the significance of catalase activity in cell response to UVA damage, experiments triggering inhibition of catalase were designed. To this end, we used a specific inhibitor of catalase, 3-amino 1,2,4-triazole (AT), that irreversibly inactivates the catalytic activity [54]. This inhibitor does not affect the *de novo* synthesis of catalase [55].

**Figure 8:**
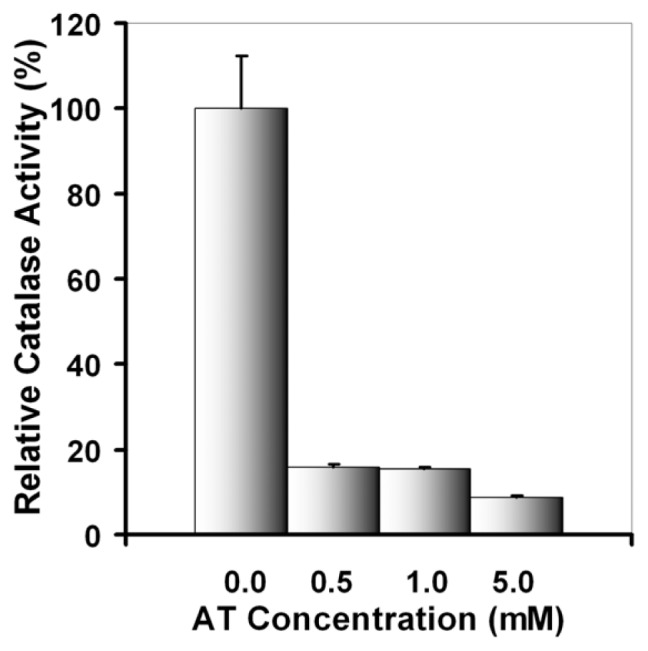
The effect of treatment with aminotriazole (AT) on catalase activity in DOK cells. Bars represent the percent of catalase activity relative to control (average ± SD, for three experiments).

To set up the experiments, the effect of three different concentrations of AT (0.5, 1.0 and 5.0 mM) on catalase activity in DOK cells was investigated. AT significantly inhibited catalase activity (Figure 8). In all subsequent experiments, a 1 mM concentration of AT was used, in accordance with the usual concentration mentioned in the literature [42,56,57]. In our experiments, this concentration of AT has decreased catalase activity in non-irradiated keratinocytes by about 85%.

**Figure 9:**
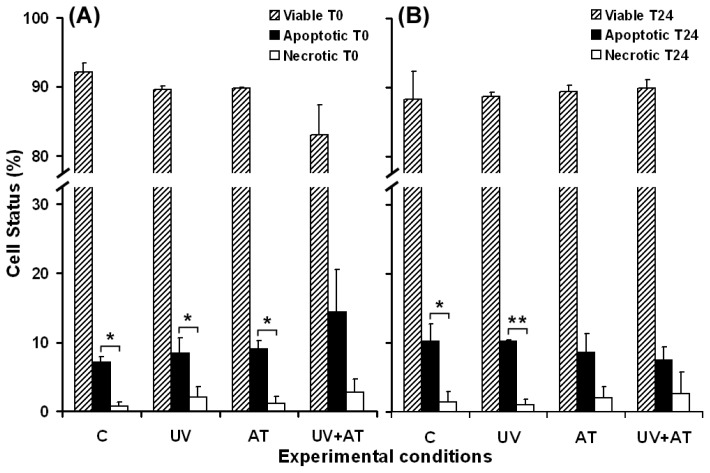
Flow cytometry data regarding cell viability, apoptosis and necrosis induced in DOK cells by 1 h UVA exposure with or without 1 mM aminotriazole (AT) treatment. (**A**) apoptosis and necrosis just after treatment; (**B**) apoptosis and necrosis in surviving DOK cells at 24 h post treatment. C—mock-irradiated cells; UV—irradiated cells; AT—cells treated with AT; UV + AT—cells treated with AT and UVA irradiated. Bars represent the percent of gated cells in three experiments; ^*^p < 0.05; ^**^p < 0.01.

The exposure of cultured keratinocytes to UVA radiation can lead to cell death, depending on the irradiation intensity and exposure time [18,58]. In our experiments, the effects of catalase inhibition with AT were examined on UVA-irradiated keratinocytes in relation to cell death. AT was added in the culture medium 1.5 h prior to cell irradiation and was removed just before UVA exposure. The flow cytometry results, regarding the analysis of DOK cell apoptosis *versus* necrosis, were recorded in four experimental conditions: (i) mock irradiated cells as control, (ii) cells without AT treatment, UVA irradiated, (iii) AT treated cells, without UVA exposure and (iv) AT treated cells, UVA irradiated. The results showed that UVA irradiation or catalase inhibition have slightly increased apoptosis and necrosis, immediately after the treatments (Figure 9A). The effect of AT and UVA was cumulative for apoptosis and necrosis (Figure 9A). The level of apoptosis of surviving cells, maintained in culture for 24 h, is similar (for UVA irradiated cells) or lower (for AT, and UVA + AT treated cells) than that of the control cells (Figure 9B). A decrease in apoptosis was noted for the cells cultured for 24 h after the combined action of stressors (Figure 9A,B), compared to the level determined just after the treatments. No significant differences were noted in terms of necrosis at 24 h post-treatments for cells in all the experimental conditions, compared to the effects at time zero (Figure 9A,B).

DOK cells carry a partially transformed phenotype that expresses increased levels of a mutant p53, harboring a 12 bp in-frame deletion (codons 188–191) [59,60]. This protein is a key player in the cell response to various stressors, including UVA [61–63]. Our results show a putative effect of UVA irradiation and/or catalase inhibition on cell death, immediately after exposure, and suggest a selection of cells gaining an adaptive surviving response. Thus, we may assume that the mechanism linking oxidative stress and p53-mediated apoptosis, which is effective in normal cells, is disturbed in dysplastic keratinocytes, leading to a low level of apoptosis when faced with a powerful oxidative stress, induced by UVA exposure, associated with catalase inhibition.

Oxidative stress is a frequent trigger of apoptosis in a variety of cells and has been proven to be a component of common apoptosis pathways [64,65]. Apoptosis was associated with peroxidation of specific phospholipids in the plasma membrane, including phosphatidylserine. Moreover, phosphatidylserine oxidation may mediate its transfer on the external leaflet of the membrane bilayer [66], a process which has been identified as one of the early and prominent events of apoptosis [67]. The results in this study lead us to consider that UVA-induced lipid peroxidation in the plasma membrane of DOK cells acts as a component of the apoptotic process, contributing to phosphatidylserine flopping. This assumption is consistent with our flow cytometry results, which confirm an increase in the number of annexin V-positive (apoptotic) cells, after UVA exposure (Figure 9A). These findings demonstrate that the plasma membrane is a cellular target of UVA and that its damage is related to oxidative processes and cell fate.

### 2.4. Adaptive Response of DOK Cells to UVA Radiation

Our results suggest the progress of an adaptive response under low stress, showing the ability of irradiated DOK cells to swiftly restore the level of polyunsaturated fatty acids affected by UVA-induced peroxidation (Figure 3, at 6 h post-irradiation). In our experimental conditions, the cells that developed such adaptive mechanisms survived, despite the presence in the culture medium of significantly increased levels of TBARS (Figure 1). The cell ability to adapt and survive after low stress is supported by the flow cytometry results that show no significant change of either apoptosis or necrosis for the surviving cell population (Figure 9). Moreover, the survival is accompanied and supported by the tendency of membrane permeability to regain its normal status (Figure 5).

More than just a target, the membrane acts as a sensor of UVA irradiation in activation of signal transduction pathways overlapping with those induced by oxidant agents [68]. UVA radiation is absorbed by several target molecules relevant for cellular signaling, stimulating various signal transduction pathways [69–71]. Combined actions of these pathways determine the genetic program that establishes the fate of UVA irradiated cells by inducing the transcription of various genes [17,72,73]. It is expected for these pathways to activate transcription of antioxidant enzymes, such as catalase, in order to protect the cells against UVA-induced oxidative stress.

In summary, our results show an oxidative stress on DOK cells induced by UVA radiations affecting both cell membrane (biochemistry, integrity and function) and antioxidant enzyme activity. Dysplastic keratinocytes exhibit resistance to apoptosis/necrosis in response to UVA exposure or catalase inhibition, and only the cumulative action of the stress factors affects cell viability, in terms of increased apoptosis and necrosis as an immediate cell response. Moreover, even under these stressing conditions, the surviving cells seem to gain an increased resistance to apoptotic death, suggesting that the pre-malignant status of these cells harbors an abnormal ability to control the cell fate.

## 3. Experimental Section

### 3.1. Cell Culture

Human Caucasian dysplastic oral keratinocytes (DOK, ECACC No. 94122104) were cultured in Dulbecco Modified Eagle’s Medium (DMEM) supplemented with Ham F12 (3:1), 10% fetal calf serum, 2 mM l-glutamine and antibiotic-antimycotic solution. Cells were seeded at a 10^4^ cells/cm^2^ density in 60 mm plastic Petri dishes and grown to 80% confluence. Cell viability was assessed using Trypan Blue exclusion assay and exceeded 95% in each experiment. Oral keratinocytes are considered to share structural and functional features with skin keratinocytes. Therefore, they offer an appropriate model for the study of keratinocyte behavior in cell culture [74].

### 3.2. UVA Source and Irradiation Conditions

Irradiation was carried out with an UV-365 nm lamp, VL-340 BLB model (Vilber Lourmat, France) at a light intensity of 381 μW/cm^2^. Prior to irradiation, cells were washed twice with 2 mL phosphate-buffered saline (PBS). Cell layers were covered with 2 mL PBS and irradiated from the top at a distance of 10 cm. Irradiation was performed for 1 or 2 h, which resulted in accumulated doses of 18.7 J/cm^2^ and 37.4 J/cm^2^, respectively, as measured [75,76] with a LaserStar Power Meter, provided with a 3A-P photodetector (Ophir Optronics Solutions Ltd, Jerusalem, Israel). To avoid thermal stimulation, UVA exposure was done in a well-ventilated laminar flow hood (Safeflow 1.8, Bioair, Siziano, Italy). The control cells (referred to as mock irradiated cells) were similarly handled, but were shielded from UVA with an aluminum foil sheet.

### 3.3. TBARS Assay

Supernatants (1 mL aliquots) were collected just after irradiation, but also at 6, 12 and 24 h post-irradiation, in order to assess lipid peroxidation. The peroxidation products were measured via thiobarbituric acid color reaction for malondialdehyde (MDA), according to Ohkawa *et al.*[77], using tetraethoxypropane for calibration, a reagent quantitatively yielding the MDA-thiobarbituric acid adduct.

### 3.4. Fatty Acid Gas Chromatography

Fatty acids in cellular lipids were simultaneously extracted and transesterified to methyl esters. Samples were dispersed in hexane and were treated with 2M potassium hydroxide in methanol (100 μL solution/100 μg cell sediment). After drying the mixture with anhydrous sodium sulphate and neutralizing potassium hydroxide with monosodium phosphate, the samples were centrifuged to obtain a clear supernatant. The quantitatively collected supernatant was concentrated under nitrogen using a Techne DB-2A concentrator (Barloworld Scientific, Burlington, NJ, USA) and a N2-Bora nitrogen generator (LNI Schmidlin AG, Neuheim, Switzerland). The methyl ester concentrates were analyzed by gas chromatography on a capillary column TR-FAME (120 m × 0.25 mm id × 0.25mm, Thermo Fischer Scientific Inc., Waltham, MA, USA), mounted in a CP-3800 gas chromatograph (Varian, Palo Alto, CA, USA) equipped with a flame ionization detector. The fatty acids were identified by comparison with retention times of standards in Supelco 37 Component FAME Mix (Sigma-Aldrich, Taufkirchen, Germany).

### 3.5. LDH Release Assay

The membrane integrity for control and irradiated cells was evaluated by quantifying the LDH release, using the Cytotox96^®^ Non-Radioactive Cytotoxicity Assay kit (Promega, Mannheim, Germany). The results were expressed as optical density (OD_492 nm_ of samples − OD_492 nm_ of blank).

### 3.6. Propidium Iodide Staining

Cell viability and changes in membrane permeability were assessed, by 5 μg/mL propidium iodide staining in PBS, for 15 min. The cell layer was washed twice in 2 mL PBS and examined under an inverted microscope in phase contrast and fluorescence.

### 3.7. Catalase Activity Assay

Cells were scraped just after irradiation in 500 μL ultrapure water (Direct Q 3, Millipore, Billerica, MA, USA) and lysed by six cycles of frost-defrost. Catalase activity in the cell lysate was measured by monitoring the disappearance of H_2_O_2_ at 240 nm using the spectrophotometric procedure described by Aebi [78]. Activities were normalized to the cell protein content, measured by the method of Lowry *et al.*[79]. To analyze the effect of catalase inhibition on cell behavior after UVA exposure, aminotriazole (AT) was used at the effective concentration of 1 mM, proven to be effective in our experimental conditions and usually used for catalase inhibition experiments. Cell incubation with AT was done for 1.5 h prior to the irradiation step, and the inhibitor was removed just before cell exposure to UVA.

### 3.8. Electrophoresis and Western Blotting

SDS-PAGE (10% gels) was performed at 20 mA/gel using the Mini-PROTEAN 3 system (Bio Rad Laboratories, Hercules, CA, USA) for catalase immunodetection. Proteins were transferred on PVDF membranes for 1 h, at 100 V, using the Mini Trans-Blot system (Bio Rad Laboratories, Hercules, CA, USA). Membranes were blocked with 5% non-fat milk in TBST (10 mM Tris-HCl, pH 8.0, 150 mM NaCl, 0.1% Tween 20), overnight, at 4 °C. Detections were carried out as follows: primary antibodies (goat polyclonal, SC-34280), 1:200 diluted in TBST, supplemented with 2% bovine serum albumin (TBST-BSA), for 2 h, at room temperature (RT), followed by appropriate HRP-conjugated secondary antibodies (donkey anti-goat IgG-HRP, CS-2020), 1:10,000 diluted in TBST-BSA, for 1 h, at RT. All antibodies were purchased from Santa Cruz Biotechnology (Santa Cruz, CA, USA). Membranes were processed by enhanced chemiluminiscence using SuperSignal^®^ Chemiluminiscent Substrate (Pierce, Rockford, IL, USA), and the signals were recorded on XCL-XPosure films (Pierce Biotechnology, Inc. Rockford, IL, USA).

### 3.9. Flow Cytometry Assay

The flow cytometric analysis was carried out using a FACScan (Becton Dickinson, San Jose, CA, USA) flow system, after annexinV-FITC/propidium iodide labeling of the cells. Cells exposed or not to UVA were prepared for flow cytometry using AnnexinV-FITC Apoptosis Detection Kit (Sigma, Saint Louis, MO, USA) according to the producer’s protocol. Before labeling, adherent and detached cells were collected and pooled, then counted and investigated for viability using the trypan blue exclusion test.

### 3.10. Statistical Analysis

Data are mean ± SD of triplicate measurements from three to five independent experiments. The statistical significance was assessed by Student *t*-test. The correlation between lipid peroxidation and LDH release (membrane lysis) was determined by Pearson’s coefficient.

## 4. Conclusions

Our results show that dysplastic keratinocytes proved the ability to develop adaptive responses after non-lethal UVA exposure, even though an oxidative stress was noted that transiently affected the balance in cell lipids fatty acids, cell membrane permeability and catalase activity. The surviving cells seemed to gain an increased resistance to apoptotic death, suggesting that the pre-malignant status of these cells could harbor an abnormal ability to control the cell fate.

## References

[1] Krutmann J. (2000). Ultraviolet A radiation-induced biological effects in human skin: Relevance for photoaging and photodermatosis. J. Dermatol. Sci.

[2] Honda A., Abe R., Makino T., Norisugi O., Fujita Y., Watanabe H., Nishihira J., Jwakura Y., Yamagishi S., Shimizu H., Shimizu T. (2008). Interleukin-1β and macrophage migration inhibitory factor (MIF) in dermal fibroblasts mediate UVA-induced matrix metalloproteinase-1 expression. J. Dermatol. Sci.

[3] Onoue S., Nonaka R., Sato F., Koide C., Hayashi A., Wachi H. (2009). Involvement of reactive oxygen species in abnormal tropoelastin deposition induced by UVA-photosensitizers. J. Health Sci.

[4] He Y.Y., Pi J., Huang J.L., Diwan B.A., Waalkes M.P., Chignell C.F. (2006). Chronic UVA irradiation of human HaCaT keratinocytes induces malignant transformation associated with acquired apoptotic resistance. Oncogene.

[5] Jans J., Garinis G.A., Schul W., van Oudenaren A., Moorhouse M., Smid M., Sert Y.G., van der Velde A., Rijksen Y., de Gruijl F.R. (2006). Differential role of basal keratinocytes in UV-induced immunosuppression and skin cancer. Mol. Cell Biol.

[6] Brash D.E., Heffernan T.P., Nghiem P, Farage M.A., Miller K.W. (2010). Carcinogenesis: UV Radiation. Textbook of Aging Skin.

[7] Sage E., Girard P.M., Francesconi S. (2012). Unravelling UVA-induced mutagenesis. Photochem. Photobiol. Sci.

[8] Wickelgren I. (2007). Skin biology: A healthy tan?. Science.

[9] Nishimura H., Yasui H., Sakurai H. (2006). Generation and distribution of reactive oxygen species in the skin of hairless mice under UVA: Studies on *in vivo* chemiluminescent detection and tape stripping methods. Exp. Dermatol.

[10] Valencia A., Kochevar I.E. (2008). Nox1-based NADPH oxidase is the major source of UVA-induced reactive oxygen species in human keratinocytes. J. Invest. Dermatol.

[11] Dalle Carbonare M., Pathak M.A. (1992). Skin photosensitizing agents and the role of reactive oxygen species in photoaging. J. Photochem. Photobiol. B.

[12] Wondrak G.T., Jacobson M.K., Jacobson E.L. (2006). Endogenous UVA-photosensitizers: Mediators of skin photodamage and novel targets for skin photoprotection. Photochem. Photobiol. Sci.

[13] Yu H., Xia O., Yan J., Herreno-Saenz D., Wu Y.S., Tanq I.W., Fu P.P. (2006). Photoirradiation of polycyclic aromatic hydrocarbons with UVA light—A pathway leading to the generation of reactive oxygen species, lipid peroxidation and DNA damage. Int. J. Environ. Res. Public Health.

[14] Herrling T., Fuchs J., Rehberg J., Groth N. (2003). UV-induced free radicals in the skin detected by ESR spectroscopy and imaging using nitroxides. Free Radic. Biol. Med.

[15] Sakurai H., Yasui H., Yamada Y., Nishimura H., Shigemoto M. (2005). Detection of reactive oxygen species in the skin of live mice and rats exposed to UVA light: A research review on chemiluminescence and trials for UVA protection. Photochem. Photobiol. Sci.

[16] Baier J., Maisch T., Maier M., Engel E., Landthaler M., Baumler W. (2006). Singlet oxygen generation by UVA light exposure of endogenous photosensitizers. Biophys. J.

[17] Allen R.G., Tresini M. (2000). Oxidative stress and gene regulation. Free Radic. Biol. Med.

[18] Assefa Z., van Laethem A., Garmyn M., Agostinis P. (2005). Ultraviolet radiation-induced apoptosis in keratinocytes: On the role of cytosolic factors. Biochim. Biophys. Acta.

[19] Kurita M., Shimauchi T., Kobayashi M., Atarashi K., Mori K., Tokura Y. (2007). Induction of keratinocyte apoptosis by photosensitizing chemicals plus UVA. J. Dermatol. Sci.

[20] Schneider L.A., Dissemond J., Brenneisen P., Hainzl A., Briviba K., Wlaschek M., Scharffetter-Kochanek K. (2006). Adaptive cellular protection against UVA-1-induced lipid peroxidation in human dermal fibroblasts shows donor-to-donor variability and is glutathione dependent. Arch. Dermatol. Res.

[21] Leccia M.T., Yaar M., Allen N., Gleason M., Gilchrest B.A. (2001). Solar simulated irradiation modulates gene expression and activity of antioxidant enzymes in cultured human dermal fibroblasts. Exp. Dermatol.

[22] Applegate L.A., Frenk E. (1995). Cellular defense mechanisms of the skin against oxidant stress and in particular UVA radiation. Eur. J. Dermatol.

[23] Rezvani H.R., Cario-André M., Pain C., Ged C., deVerneuil H., Taïeb A. (2007). Protection of normal human reconstructed epidermis from UV by catalase overexpression. Cancer Gene Ther.

[24] Morel Y., Barouki R. (1999). Repression of gene expression by oxidative stress. Biochem. J.

[25] Bickers D.R., Athar M. (2006). Oxidative stress in pathogenesis of skin disease. J. Invest. Dermatol.

[26] Keyse S.M., Tyrrell R.M. (1989). Heme oxygenase is the major 32-kDa stress protein induced in human skin fibroblasts by UVA radiation, hydrogen peroxide and sodium arsenite. Proc. Natl. Acad. Sci. USA.

[27] Hanselmann C., Mauch C., Werner S. (2001). Haem oxygenase-1: A novel player in cutaneous wound repair and psoriasis?. Biochem. J.

[28] Sedlak T.W., Snyder S.H. (2004). Bilirubin benefits: Cellular protection by a biliverdin reductase antioxidant cycle. Pediatrics.

[29] Kawanishi S., Hiraku Y. (2001). Sequence specific DNA damage induced by UVA radiation in the presence of endogenous and exogenous photosensitizers. Curr. Probl. Dermatol.

[30] Mouret S., Baudouin C., Charveron M., Favier A., Cadet J., Douki T. (2006). Cyclobutane pyrimidine dimers are predominant DNA lesions in whole human skin exposed to UVA radiation. Proc. Natl. Acad. Sci. USA.

[31] Campalans A., Amouroux R., Bravard A., Epe B., Radicella J.P. (2007). UVA irradiation induces relocalisation of the DNA repair protein hOGG1 to nuclear speckles. J. Cell Sci.

[32] Marrot L., Meunier J.R. (2008). Skin DNA photodamage and its biological consequences. J. Am. Acad. Dermatol.

[33] Besaratinia A., Synold T.W., Xi B., Pfeifer G.P. (2004). G-to-T tranversions and small tandem base deletions are the hallmark of mutations induced by ultraviolet A radiations in mammalian cells. Biochemistry.

[34] Huang X.X., Bernerd F., Halliday G.M. (2009). Ultraviolet A within sunlight induces mutations in the epidermal basal layer of engineered human skin. Am. J. Pathol.

[35] Mitchell D. (2006). Revisiting the photochemistry of solar UVA in human skin. Proc. Natl. Acad. Sci. USA.

[36] Molho-Pessach V., Lotem M. (2007). Ultraviolet radiation and cutaneous carcinogenesis. Curr. Probl. Dermatol.

[37] Ibbotson S.H., Lambert C.R., Moran M.N., Lynch M.C., Kochevar I.E. (1998). Benzoyl peroxide increases UVA-induced plasma membrane damage and lipid oxidation in murine leukemia L1210 cells. J. Invest. Dermatol.

[38] Gniadecki R., Christoffersen N., Wulf H.C. (2002). Cholesterol-rich plasma membrane domains (lipid rafts) in keratinocytes: Importance in the baseline and UVA-induced generation of reactive oxygen species. J. Invest. Dermatol.

[39] Pandey B.N., Mishra K.P. (2003). *In vitro* studies on radiation induced membrane oxidative damage in apoptotic death of mouse thymocytes. Int. J. Low Radiat.

[40] Larsson P., Anderson E., Johansson U., Öllinger K., Rosdahl I. (2005). Ultraviolet A and B affect human melanocytes and keratinocytes differently. A study of oxidative alterations and apoptosis. Exp. Dermatol.

[41] Budai M., Reynaud-Angelin A., Szabó Z., Tóth S., Rontó G., Sage E., Gróf P. (2004). Effect of UVA radiation on membrane fluidity and radical decay in human fibroblasts as detected by spin labeled stearic acids. J. Photochem. Photobiol. B.

[42] Vile G.F., Tyrrell R.M. (1995). UVA radiation-induced oxidative damage to lipids and proteins *in vitro* and in human skin fibroblasts is dependent on iron and singlet oxygen. Free Radic. Biol. Med.

[43] Armeni T., Damiani E., Battino M., Greci L., Principato G. (2004). Lack of *in vitro* protection by a common sunscreen ingredient on UVA-induced cytotoxicity in keratinocytes. Toxicology.

[44] Polte T., Tyrrell R.M. (2004). Involvement of lipid peroxidation and organic peroxides in UVA-induced matrix metalloproteinase-1 expression. Free Radic. Biol. Med.

[45] Girotti A.W. (2001). Photosensitized oxidation of membrane lipids: Reaction pathways, cytotoxic effects, and cytoprotective mechanisms. J. Photochem. Photobiol. B.

[46] Hoerter J.D., Ward C.S., Bale K.D., Gizachew A.N., Graham R., Reynolds J., Ward M.E., Choi C., Kagabo J.L., Sauer M. (2008). Effect of UVA fluence rate on indicators of oxidative stress in human dermal fibroblasts. Int. J. Biol. Sci.

[47] Doherty G.J., McMahon H.T. (2008). Mediation, modulation, and consequences of membrane-cytoskeleton interactions. Annu. Rev. Biophys.

[48] Goyal M.M., Basak A. (2010). Human catalase: Looking for complete identity. Protein Cell.

[49] Aronov S. (1965). Catalase kinetics of photooxidation. Science.

[50] Gantchev T.G., van Lier J.E. (1995). Catalase inactivation following photosensitization with tetrasulfonated metallophtalocyan. Photochem. Photobiol.

[51] Aubailly M., Haigle J., Giordani A., Morlière P., Santus R. (2000). UV photolysis of catalase revised: A spectral study of photolytic intermediates. J. Photochem. Photobiol. B.

[52] Zigman S., Reddan J., Schultz J.B., McDaniel T. (1996). Structural and functional changes in catalase induced by near-UV radiation. Photochem. Photobiol.

[53] Maresca V., Flori E., Briganti S., Camera E., Cario-André M., Taïeb A., Picardo M. (2006). UVA-induced modification of catalase charge properties in the epidermis is correlated with the skin phototype. J. Invest. Dermatol.

[54] Margoliash E., Novogrodsky A., Schejter A. (1960). Irreversible reaction of 3-amino-1,2,4-triazole and related inhibitors with the protein of catalase. Biochem. J.

[55] Lubinsky S., Bewley G. (1979). Genetics of catalase in *Drosophila melanogaster*: Rates of synthesis and degradation of the enzyme in flies aneuploid and euploid for the structural gene. Genetics.

[56] Courgeon A.M., Rollet E., Becker J., Maisonhaute C., Best-Belpomme M. (1988). Hydrogen peroxide (H_2_O_2_) induces actin and some heat-shock proteins in Drosophila cells. Eur. J. Biochem.

[57] Batandier C., Fontaine E., Keriel C., Leverve X.M. (2002). Determination of mitochondrial reactive oxygen species: Methodological aspects. J. Cell. Mol. Med.

[58] Shorrocks J., Paul N.D., McMillan T.J. (2008). The dose rate of UVA treatment influences the cellular response of HaCaT keratinocytes. J. Invest. Dermatol.

[59] Chang S.E., Foster S., Betts D., Marnock W.E. (1992). DOK, a cell line established from human dysplastic oral mucosa, shows a partially transformed non-malignant phenotype. Int. J. Cancer.

[60] Burns J.E., Clark L.J., Yeudall W.A., Mitchell R., Mackenzie K., Chang S.E., Parkinson E.K. (1994). The p53 status of cultured human premalignant oral keratinocytes. Br. J. Cancer.

[61] De Laat A., Kroon E.D., de Gruijl F.R. (1997). Cell cycle effects and concomitant p53 expression in hairless murine skin after longwave UVA (365 nm) irradiation: A comparison with UVB irradiation. Photochem. Photobiol.

[62] Zhang H. (2006). p53 plays a central role in UVA and UVB induced cell damage and apoptosis in melanoma cells. Cancer Lett.

[63] Sun W., Yang J. (2010). Functional mechanisms for human tumor suppressors. J. Cancer.

[64] Kim K.W., Ha K.Y., Lee J.S., Rhyu K.W., An H.S., Woo Y.K. (2007). The apoptotic effects of oxidative stress and antiapoptotic effects of caspase inhibitors on rat notochordal cells. Spine.

[65] Ott M., Gogvadze V., Orrenius S., Zhivotovsky B. (2007). Mitochondria, oxidative stress and cell death. Apoptosis.

[66] Kagan V.E., Fabisiak J.P., Shvedova A.A., Tyurina Y.Y., Tyurin V.A., Schor N.F., Kawai K. (2000). Oxidative signaling pathway for externalization of plasma membrane phosphatidylserine during apoptosis. FEBS Lett.

[67] Verhoven B., Schlegel R.A., Williamson P. (1995). Mechanisms of phosphatidylserine exposure, a phagocyte recognition signal, on apoptotic T lymphocytes. J. Exp. Med.

[68] Bender K., Blattner C., Knebel A., Iordanov M., Herrlich P., Rahmsdorf H.J. (1997). UV-induced signal transduction. J. Photochem. Photobiol. B.

[69] Le Panse R., Dubertret L., Coulomb B. (2003). p38 mitogen-activated protein kinase activation by ultraviolet A in human dermal fibroblasts. Photochem. Photobiol.

[70] Grether-Beck S., Timmer A., Felsner I., Brenden H., Brammertz D., Krutmann J. (2005). Ultraviolet A-induced signaling involves a ceramide-mediated autocrine loop leading to ceramide *de novo* synthesis. J. Invest. Dermatol.

[71] Krutmann J. (2006). The interaction of UVA and UVB wavebands with particular emphasis on signalling. Prog. Biophys. Mol. Biol.

[72] Silvers A.L., Bowden G.T. (2002). UVA irradiation-induced activation of activator protein-1 is correlated with induced expression of AP-1 family members in the human keratinocyte cell line HaCaT. Photochem. Photobiol.

[73] Yu L., Venkataraman S., Coleman M.C., Spitz D.R., Wertz P.W., Domann F.E. (2006). Glutathione peroxidase-1 inhibits UVA-induced AP-alpha expression in human keratinocytes. Biochem. Biophys. Res. Commun.

[74] ScienCell Research Laboratories Human Oral Keratinocytes.

[75] Gavrila C., Gruia I., Lungu C.P. (2011). Optical emission phenomena modeling in TVA carbon plasma. Adv. Appl. Plasma Sci..

[76] Gavrila C., Gruia I., Lungu C.P. (2009). Determining the radial distribution of the emission coefficient from a plasma source. Optoelecron. Adv. Mater.

[77] Ohkawa H., Ohishi N., Yagi K. (1979). Assay for lipid peroxides in animal tissues by thiobarbituric acid reaction. Anal. Biochem.

[78] Aebi H, Packer L. (1984). Catalase *In Vitro*. Methods in Enzymology.

[79] Lowry O.H., Rosebrough N.J., Farr A.L., Randall R.J. (1951). Protein measurement with the Folin phenol reagent. J. Biol. Chem.

